# Effect of adipose derived stromal vascular fraction on leprosy neuropathy: A Preliminary report

**DOI:** 10.1371/journal.pntd.0010994

**Published:** 2023-01-03

**Authors:** Sondang P. Sirait, Kusmarinah Bramono, Sri Linuwih Menaldi, Jeanne Adiwinata Pawitan, Wresti Indriatmi, Tiara Aninditha

**Affiliations:** 1 Dermatovenerology Department, Faculty of Medicine, Universitas Indonesia/Dr. Cipto Mangunkusumo General Hospital, Jakarta, Indonesia; 2 Department of Histology, Faculty of Medicine, Universitas Indonesia, Jakarta, Indonesia; 3 Stem Cells Medical Technology Integrated Service Unit, Dr. Cipto Mangunkusumo General Hospital/Faculty of Medicine, Universitas Indonesia, Jakarta, Indonesia; 4 Stem Cells and Tissue Engineering Research Center, Indonesia Medical Education and Research Institute (IMERI), Faculty of Medicine, Universitas Indonesia, Jakarta, Indonesia; 5 Neurology Department, Faculty of Medicine, Universitas Indonesia/Dr. Cipto Mangunkusumo General Hospital, Jakarta, Indonesia; Hospital Infantil de Mexico Federico Gomez, MEXICO

## Abstract

**Background:**

Adipose derived stromal vascular fraction (SVF) contains a heterogeneous population of mononuclear cells, progenitor cells and about 1–10% are mesenchymal stromal cells. These cells are an ideal candidate for regenerative medicine for peripheral neuropathy. Leprosy is a disabling disorder with neuropathy, usually with consequences of permanent disability of the extremities. We conducted a preliminary study to evaluate the cell yield, its characteristics and clinical outcomes after SVF injections in four leprosy patients.

**Methods:**

Four post leprosy patients were recruited and evaluated for sensory testing (warm detection, cold detection, vibration, pain and sensation) on the ulnar area of the hand. Liposuction was done and adipose tissue was processed into SVF with a closed system and injected to the ulnar area of the hand at the dorsal and palmar side. Evaluation of sensory testing was done after 3 days, 1 week, 1 month and 3 months following SVF injection. SVF was also characterized using flow cytometry, cell counting, sterility and presence of mycobacteria.

**Results:**

The results showed that leprosy patients had a low count of mesenchymal cells and a high amount of CD34/CD45 positive cells. One patient was positive for mycobacteria from his adipose tissue and SVF. Sensory examination after SVF injection showed an improvement in temperature and pain sensation in the palmar and superficial branch. Meanwhile, touch sensation improved on the dorsal branch, and there was no improvement for vibration in all patients.

**Conclusions:**

The results showed that SVF had a potential to improve sensory loss in leprosy patients.

## Author summary

Leprosy is an infectious disease caused by *M*. *leprae* bacteria. Generally, this disease infects the skin and peripheral nerves. Most of the leprosy cases treatment is delayed, which causes nerve impairment and permanent disability. Sensory ulnar nerve impairment is the most common disability found in leprosy. Earliest signs that could be observed are paresthesia or anesthesia and unable to perceive other sensations. Permanent disability in leprosy cases usually leads to negative stigma and isolation of the patients. Thus, the treatment for nerve impairment has been one of the WHO global leprosy strategy. Adipose tissue were known as source of stem cells and could be processed to be used as a injected treatment, in the form of stromal vascular fraction (SVF). These cells could develop into many kinds of cells, including nerve cells, while also repairing the impaired cells. However, currently there are no available studies on SVF treatment for nerve impairment caused by leprosy. Therefore, this preliminary study aims to determine whether SVF cells of leprosy patients could be used as treatment for nerve impairment caused by leprosy. We report 4 post leprosy patients who underwent SVF injection intradermally on their ulnar area of the hand, with promising results.

## Introduction

The use of adipose-derived progenitors as a therapeutic agent has grown substantially in the past decade. Adipose derived stromal vascular fraction (SVF) obtained from healthy subjects is a promising alternative to more expensive and time consuming culture procedure of stem cells. The safety use of autologous SVF has been documented in several studies [1] [2],. In previous trials, the characteristic of the cells in SVF varies substantially, with a wide range of viability. The SVF contains a higher percentage of stromal cells, although multiple other lineages, most notably those of endothelial, hematopoietic and pericytic origin, are also present. When SVF cells are seeded into culture, a subset of elongated cells begins to adhere to the tissue culture plasticware. This process allows the emergence of an adherent cell population that is termed adipose tissue-derived stromal cells (ADSCs) [3]. The use of SVF from patients with chronic diseases such as scleroderma and diabetes have been documented, but until now there has been no report of SVF from leprosy patients [2] [4],^.^Leprosy is a disabling disorder with neuropathy, usually with consequences of permanent disability of the extremities. Previous study by Van Brakel, et al had showed that 48% of newly found cases without any disabilities on the initial diagnosis could progressed into disabilities five years later [5]. We hypothesize that SVF is a potential treatment for peripheral neuropathy in leprosy patients.

## Methods

### Ethics statement

The study aim was to extract and assess SVF from leprosy subjects, inject it into the sensory ulnar area of the hand and measure the clinical outcomes. Four released from treatment leprosy subjects were recruited, which all had different types of multibacillary leprosy. This study protocol was approved by the Health Research Ethics Committee, Faculty of Medicine, Universitas Indonesia and Cipto Mangunkusumo Hospital (No. 59/UN2.F1/ETIK/2018). All the subjects has signed an informed consent statement that stated they have received and understood the procedures given in this study, and willing to participate in every steps of the study.

### Study methods

Adipose tissue was taken by a mini-liposuction on the abdominal area with a Mercedes cannula manually under tumescent anesthesia. Adipose tissue was collected in two or more 50 ml syringes and transported to the laboratory for SVF extraction. Fat was transferred to a sterile collection bag without contact with air using a luer lock to luer lock connector. Further, 0.15% Collagenase solution was added and incubated for 1 hour. Using a single use kit CS-900.2 the incubated adipose tissue was connected to a Sepax 2, which was an adipose extraction device for a fully automated cellular separation. Twelve milliliters of SVF was collected in a sterile 50 ml syringe.

Samples were taken for endotoxin testing using Lonza kit, and contamination assessments using Mycoplasma kit to detect Mycoplasma, and blood agar for bacterial culture. The calculation of viable stromal cells was done using an automated cell counter (Countess II) with trypan blue staining. Cell characterization was done by flow cytometry using mesenchymal and hematological markers. Freshly isolated SVF was analyzed for surface marker expression using a FACS Canto flow cytometer analyzer (BD System). The primary antibodies used were CD34 antibodies-FITC (fluorescein isothiocyanate) and CD45 antibodies–PerCP (peridinin chlorophyll protein) (BD, San Diego, California), and human MSC analysis kit (BD, San Diego), which contained CD90 antibodies—FITC, CD73 antibodies—APC (allophycocyanin), CD105 antibodies—PerCP and Lineage negative antibodies—PE (phycoerythrin).

Detection of *M*. *leprae* DNA in the blood, adipose tissue, and SVF was carried out by obtaining the samples from each patients through mini-liposuction. The samples were immediately transported at room temperature to the laboratory within 4 hours, stored at 2–8° C, and processed within 12 hours. To obtain total DNA, the SVF and adipose tissue samples were centrifuged at 12,000 rpm for 5 minutes, and the pellet DNA was extracted by using QIAamp DNA Mini Kit (Qiagen, Hilden, Germany) according to manufacturer’s instruction to yield 40 μl of final elution. For whole blood, an amount of 200-μl whole blood was extracted by using High Pure PCR Template Preparation Kit (Roche Diagnostic GmbH, Germany) according to manufacturer’s instruction to yield 70 μl of final elution.

Primers (5’-GCA GTA TCG TGT TAG TGA A-3’ and 5’-CGC TAG AAG GTT GCC GTA TG-3’) and probe (FAM-TCG ATG ATC CGG CCG TCG GCG-TAMRA) were used to amplify and identify mycobacterium leprae, respectively as was reported previously [6]. The PCR was performed by the following compositions (20 μl of total reaction volume): 1x KAPA Probe Fast qPCR Master Mix (KAPA Biosystems), 0.2 μM each of primers, 0.4 μM probe, and 5 μl DNA template. The thermal cycle was performed by using Lightcycler 2.0 (Roche Diagnostic GmbH, Germany) with the following conditions: 95°C for 3 min and 45 cycles of 95°C for 15 sec and 60°C for 1 min.

**Fig 1:**
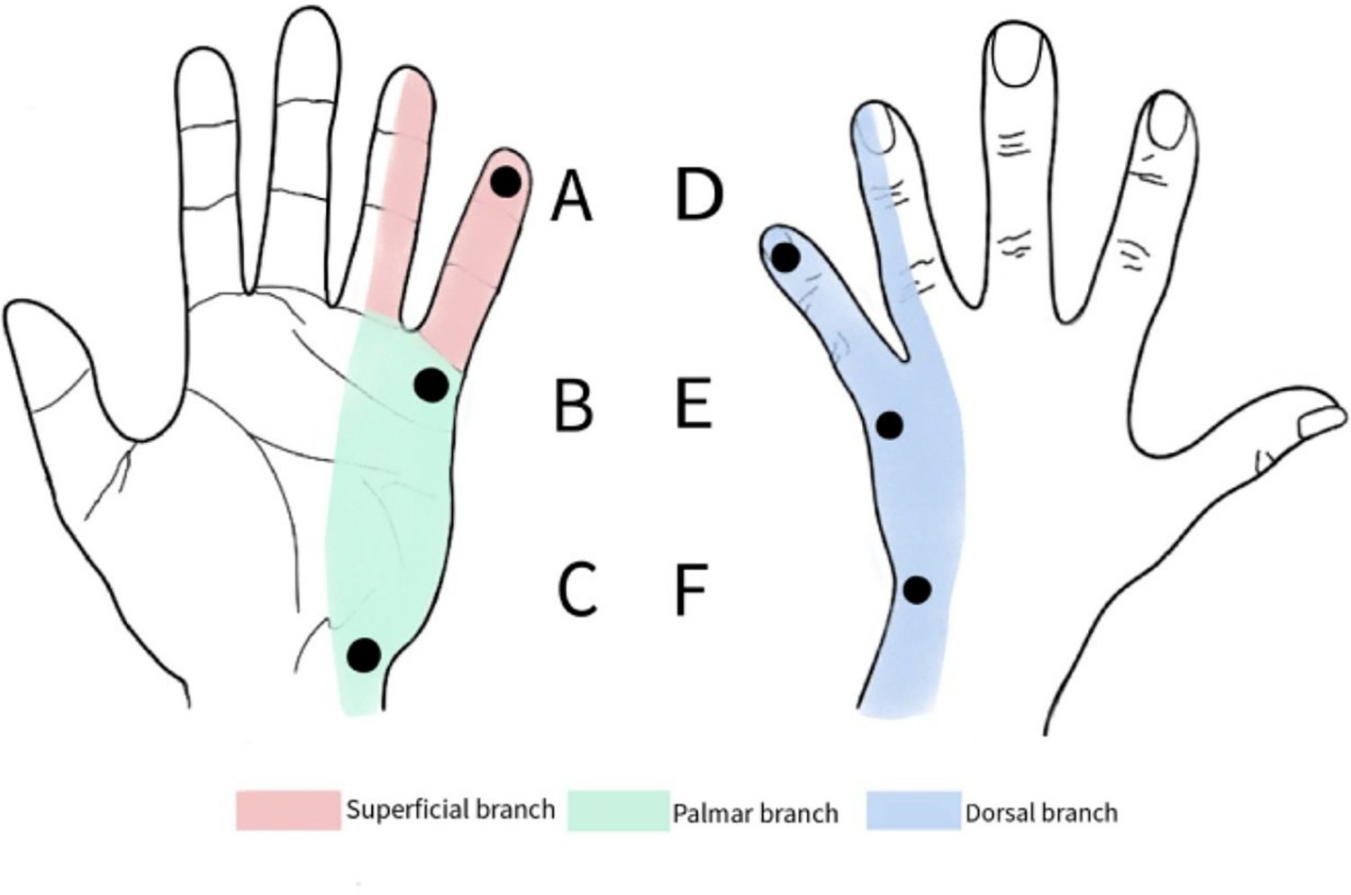
Sensory testing locations.

In this study, only sensory function was evaluated. Each leprosy patient was assessed before the SVF injection and evaluated periodically on 3^rd^ day, 1^st^ week, 1^st^ month, and 3^rd^ month following the injection. We followed the methods of The ILEP Nerve Function Impairment and Reaction (INFIR) cohort study on leprosy to evaluate sensory nerve function for touch, vibration, warm detection threshold (WDT), and cold detection threshold (CDT) [7]. We also added pain sensation to the evaluation. Sensory testing was done on several locations on the palmar and dorsal aspect of the ulnar area of the hand (Fig 1). For touch sensation, 20 Semmes Weinstein monofilament was used. WDT and CDT were assessed using two test tubes containing warm water (40°C) and cold water (20°C) that were affixed alternately. Sensitivity to pain was checked with the sharp end of a needle (pinprick) and the blunt end of a pin. The vibration test was assessed using a 128-Hz tuning fork, the examiner compared the patient’s vibrational perception threshold with his/her own as the normal control. The scores were sorted and picked the highest according to three branch of ulnar nerve (A for superficial, B and C for palmar, and D, E, and F for dorsal). Subjective neuropathic pain was assessed using painDETECT questionnaire. The results of the painDETECT questionnaire were categorized into three groups. Nociceptive pain if the painDETECT questionnaire score <12, neuropathic pain if > 19 and the value in between (13–18) is ambiguous, but neuropathic pain can still be found.

## Results

Sampling was carried out on four leprosy patients (patient 1–4), which are all male. The youngest patient was 19 years and the oldest was 61 years. The lowest Body Mass Index (BMI) was 14.75 and the highest was 33.24. Subcutaneous fat harvesting sites differed between subjects, these include lower abdomen and flank in patients with low BMI. The lowest number of cells in lipoaspirate was found in one patient aged 61 years. Other patients varied widely in numbers, ranging from 556.666 to 1.940.000 cells/ml of aspirate. The patient with obesity had the highest cell count. All of the data mentioned could be seen on Table 1.

**Table 1:** Clinical, Lipoaspirate and Cells Profile.

Patient’s Characteristics				
	Patient 1	Patient 2	Patient 3	Patient 4
Gender/Age	M / 28	M / 20	M / 61	M / 41
BMI	14.75	14.52	22	33.24
Location of *mini liposuction*	Flank	Flank	Lower Abdomen	Lower Abdomen
Total Cell Lipo-aspirated (cells)	33.4 x 10^6^	32.3 x 10^6^	24.6 x 10^6^	194 x 10^6^
Lipoaspirate (mL)	60	60	90	100
Cell / mL lipoaspirate	556.666	538.333	273.333	1.940.000
Viability (%)	63.7	67.3	63.7	61.4
Bacterial Culture	Negative	Negative	negative	negative
Mycoplasma	Negative	Negative	negative	negative
Endotoxin	Negative	Negative	negative	borderline
**Characteristics of Cells**				
CD73+ (%)	0.1	1.3	1.2	3
CD90+ (%)	0.7	6	3	5.6
CD105+ (%)	0.2	2.4	9.9	2
Lin-neg (%)	1.2	8	4.9	6.7
CD34+ (cell/μl)	14.85	8.58	26.69	21.7
CD34+ (%)	3.7	2.2	7.8	2.9
CD34+/45+ (%)	3.7	2.2	7.2	2.69

M = male, BMI = Body Mass Index, SVF = Stromal Vascular Fraction

CD = *Cluster of Differentiation*, Lin-neg = *Lineage Negative*

Culture tests on blood agar showed negative results in all patients, which meant that the process of making SVF with this method was sterile. Mycoplasma was also not found in all patients, whereas endotoxin was borderline in the fourth patient.

Patient 1 had a positive PCR result to *M*. *leprae* from unprocessed adipose tissue and SVF. Other remaining patients were PCR negative for *M*. *leprae*.

**Fig 2:**
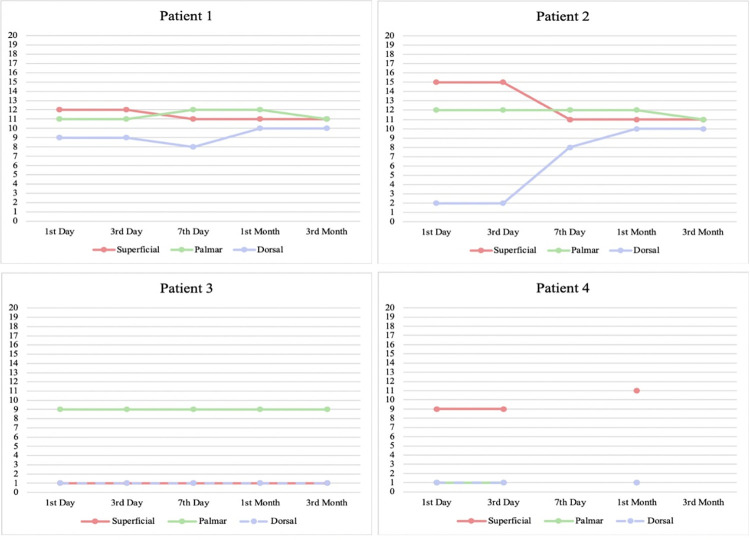
Sensation assessed with 20 Semmes Weinstein monofilament, scored 0–20 from the largest filament (180 g) to the smallest (0,008 g). As seen on the figure, Patient 1 and Patient 2 showed improvement in the dorsal branch, while Patient 4 showed improvement in the superficial branch. There was no improvement for Patient 3.

The touch sensation examination result was given score 0–20 from the largest filament (300 g) to the smallest filament (0.008 g), as seen in Fig 2. There was an improvement in touch sensation in patient 1 and 2, at the dorsal branch of the ulnar nerve. Moreover, patient 4 exhibits improvement at the superficial branch of the nerve. Patient 3 results did not differ throughout observation.

Patient 1 experienced an improvement in WDT and CDT on both superficial and palmar branch. This patient worked in a shop where he had to handle cold objects from the refrigerator. He was surprised because he could feel a cold sensation that he had not felt in a long time. On the contrary, patient 2 experienced worsening of WDT, although CDT was not affected. Patient 3 did not have any improvement in WDT and CDT. Patient 4 experienced improvement at the superficial branch for WDT. All of the sensory testing results could be seen on Table 2.

**Table 2:** Sensory testing for Warm Detection Threshold, Cold Detection Threshold, Pin prick test and Vibration test.

Pts. No.	Ulnar Nerve Branch	Sensory Testing Result											
		WDT			CDT			Pin prick			Vibration		
		B	1 mo	3 mo	B	1 mo	3 mo	B	1 mo	3 mo	B	1 mo	3 mo
1	Superficial	0	0	1	0	1	1	1	2	2	1	0	0
	Palmar	0	1	1	0	1	1	1	2	2	1	1	1
	Dorsal	0	0	0	0	0	0	1	2	0	1	1	1
2	Superficial	1	0	0	1	1	1	1	2	2	1	1	1
	Palmar	1	0	0	1	1	1	1	2	2	2	1	1
	Dorsal	1	0	0	1	1	1	1	2	1	1	1	1
3	Superficial	0	0	0	0	0	0	1	1	1	1	1	1
	Palmar	0	0	0	0	0	0	1	1	1	1	1	1
	Dorsal	0	0	0	0	0	0	0	0	1	1	1	1
4	Superficial	0	1	N/A	0	1	N/A	1	1	N/A	1	1	N/A
	Palmar	0	0	N/A	0	0	N/A	0	0	N/A	1	1	N/A
	Dorsal	0	0	N/A	0	0	N/A	0	0	N/A	1	1	N/A

Pts, No.: Patients number

WDT: warm detection threshold, CDT: cold detection threshold; B: baseline, 1 mo: 1^st^ month after injection, 3 mo: 3^rd^ month after injection

WDT and CDT scoring: 0: unable to perceive warmth/cold, 1: able to perceive warmth/cold sensation

Pin prick scoring: 0: anesthesia/unable to feel pain, 1: hipoesthesia/pain sensation reduced, 2: normal sensation

Vibration scoring: 0: unable to sense vibration, 1: vibration sensation duration shortened (compared to observer), 2: able to sense vibration normally (compared to observer)

Pinprick detection results in patient 1 and 2 improved at the superficial and palmar branch, but worsened at the dorsal branch. Patients 3 and 4 continued to have hypoesthesia at the entire ulnar area examined. Improvements in the palmar and superficial branches of the ulnar nerve correspond to improvements in the sensation of warm and cold temperatures. However, the sense of touch improvement was experienced at the dorsal ulnar branch. Vibration sensation examination did not show any improvement in all patients.

After the SVF injection, there was an improvement in subjective complaints with the PainDETECT questionnaire, neuropathic pain had decreased on the third day and persisted until day 90, except for one subject who experienced a recurrence of reversal reaction. PainDETECT questionnaire scores for each patient could be seen on Table 3.

**Table 3:** PainDETECT questionnaire scores for each patients.

Time of Examination	Patient 1	Patient 2	Patient 3	Patient 4
Before SVF Injection	20	5	23	6
Day 3 after injection	0	3	16	6
Day 7 after injection	9	3	11	N/A
1 Month after injection	9	0	18	6
3 Months after injection	16	2	14	N/A

Scoring: ≤ 12 = nociceptive pain; 13–18 = ambigous, neuropathic pain can still be found; > 19 = neuropathic pain.

## Discussion

Leprosy neuropathy is an important cause of morbidity and disability. Currently, there are no specific treatments for neuropathy. Our preliminary study will add to the armamentarium of newer treatments to overcome neuropathy.

Our study showed that the oldest patient had the lowest cell yield. Previous studies have shown adipose derived stem cells from younger group of patients have the capability to proliferate faster with higher success rate in differentiating into mature adipocytes than in older patients [8]. The capacity to regenerate blood vessels and vascularization of adipose tissue was found to be decreased with an increasing age as well. In elderly the tissue is overall less vascularized with decrease in adipose tissue cellularity. Stromal vascular cells are located in the surrounding of blood vessels and attached to the extracellular matrix, this may be related to the decrease in cell yield in older patients [9].

In this preliminary study, two subjects were severe underweight (patient 1 and 2), normal weight (patient 3), and one class 1 obese (patient 4). In obesity, the adipocytes are enlarged and have an increased distance between adipocytes and surrounding vasculature. Enlarged adipocytes can grow up to 100–200 μm, with a diameter often more than normal distance of oxygen diffusion capability into tissue. Studies have demonstrated that oxygen concentration is close to zero at 100 μm distance from the vasculature. Hypoxic environment triggers an inflammatory response in an attempt to increase blood flow and stimulate angiogenesis. However, the process is insufficient to compensate the continuous growth of adipocytes that results in chronic inflammatory process [10], accumulation of macrophage in adipose tissue and complications of metabolic syndrome [11]. In obesity and post-obesity condition, microenvironment in subcutaneous adipose tissue can change the phenotype of healthy ADSC into unhealthy ADSC. Further research needs to be done with the use of ADSC/SVF from obese/post obesity patients for regenerative therapy. In this study, total CD34+ and Lin-neg was not different between obese and non-obese patients. In adipose tissue, the population of preadipocytes can be identified by surface markers: CD45-, CD146-, CD31-, CD34+ and Lin-neg, CD34+ cells contain adipogenic progenitors [12].

Superficial abdomen fat is less susceptible to apoptotic stimulants; therefore, it is ideally used in regenerative therapy. In our study 1,940,000–2,330,000 cells / ml from the lower abdomen and from the flank 556,666–967,500 cells / ml, without eliminating the elderly patient who had less cell count. Harvesting subcutaneous fat in the lower abdomen location remains an ideal location, because it is easier to be accessed, the amount of fat collected is usually in abundance, and the cells obtained are also more.

Cell viability in this preliminary study was less than 70%. According to IFATS and ISCT recommendations, SVF cell viability should be more than 70%. Vickers et al mentioned different results on cell viability, although some were less than 70%, it still showed good treatment results [1].

In our study, there was one patient who had borderline endotoxin in the SVF. Positive endotoxins possibly caused by flawed washing process of SVF after mixing it with collagenase resulted in some collagenase residue in SVF. The criteria of good and acceptable SVF are negative for endotoxin, negative for mycoplasma, and sterile.

One leprosy patient still had mycobacteria in his adipose tissue and SVF, although already completed his multi drug treatment. This result could be due to the presence of bacilli in blood that contaminated the harvested adipose tissue or in the adipose tissue itself. This could open new discussions about pathogenesis of leprosy and where the bacteria reside. This patient also had a recurring reversal reaction which could be due to the persisting mycobacteria, although the bacteria could not be confirmed viable or dead.

Until now, CD34 is still an acceptable marker for hematopoietic stem cells due to its previous use in umbilical cord transplant. In a study, Maumus et al found that ADSC with CD34+ did not serve as a marker for in vivo pericytic process. One of the possibilities, CD34 is a marker for a different type of progenitor cells, MSC and endothelial vascular progenitor cell. The result concluded CD34 as physiological niche-specific marker for immature cells / early progenitors cells that was lost in in-vitro condition [13]. Pre-adipocyte population can be identified through flow cytometry with the use of cell surface marker: CD45-, CD146-, CD31-, CD34+. In this preliminary study, most of the CD34+ cells were also CD34+/CD45+, which are the human leukocyte antigen from the lineage of leukocyte common antigen (LCA) [14]. CD34+/CD45+ antigen is present in all human leucocyte and expressed weakly on hematopoietic progenitor cells [12]. We assumed that these results were due to a possibility of ongoing mild chronic inflammatory process in adipose tissue in leprosy patients.

CD90 which is the marker of stem cells was highest in patient 2, which showed the best improvement of sensation. We suspect because this patient is still young, he had more stem cells than the older patients. Similarly he also had a lower CD34+/CD45+ cells, which possibly could mean a lower inflammation level.

This preliminary study discovered improvement in touch sensation, especially in the dorsal ulnar branch. Improvements in pain sensation in the palmar and superficial branches of the ulnar nerve correspond to improvements in the sensation of warm and cold temperatures. Vibration sensation examination did not show improvement in all patients. Vibration of the 128 Hz tuning fork can detect mechanoreceptors, especially Paccini’s corpuscle and large nerves (A-beta). Probably these larger nerves need longer time to regenerate and will not show improvement in 3 months of this preliminary study.

In this study, patient 1 with nociceptive pain also experienced a decrease in pain scores, while patient 4 did not experience any changes. Thus, it can be concluded from the results of this study, that SVF from leprosy patients can be expected to reduce neuropathic pain and reduce nociceptive pain in the ulnar nerve. Ceceli et al. [15] showed PainDETECT had a significant correlation with VAS scores. The painDETECT scoring system is also used as an effective descriptor of neuropathic pain in describing the components of neuropathic pain and as an instrument for evaluating optimal pain management. In Indonesia, this questionnaire has been translated into Indonesian language since 2014 and is considered valid and reliable with high sensitivity and specificity in screening for the components of neuropathic pain [16].

The clinical improvement result of this study is in accordance with other studies, that freshly harvested SVF containing large numbers of CD34+ cells injected to mouse ischemic hind limb, was associated with an increase in the blood flow and the capillary density and an incorporation of the cells in the leg vasculature [17]. A study by Karatan et al found that myelin sheath becomes thicker after SVF injection [18].

This study results supports one of the goal of WHO strategy towards zero leprosy, “manage leprosy and its complications and prevent new disability” with one of the key research areas is optimized and new treatment options for reactions and nerve function impairment. As stated in the strategy, 3–4 million people with physical disabilities caused by leprosy potentially suffer from social exclusion and stigmatisation [19]. From the improvement we observed in this study, we expect that this could be a new breakthrough as a new treatment option for disability in the form of nerve function impairment to help on speeding up the process towards the ending of leprosy in 2030, specifically on reducing the rate of new cases with grade 2 disability.

We understand that this study had some limitations regarding the subject number and selection, with only four men subjects. Females generally have more adipose tissues compared to men. Therefore the results of SVF harvest and injection may differ from those in males. However, a larger study with RCT design with more inclusive subjects is ongoing as this preliminary study result considered satisfactory.

## Conclusion

SVF from leprosy patients have enough progenitor and stem cells, although MSC content was very low. Sensory sensation was improved, especially on the palmar aspect for WDT and CDT, but touch sensation was more improved on the dorsal aspect of the hand. Improvement in sensation demonstrates that SVF from leprosy patients may have potential therapeutic effects, and further research should be done to confirm their efficacy in a clinical trial. We conclude that injection of the SVF may have a positive effect on nerve regeneration in leprosy and could contribute to WHO global leprosy strategy 2021–2030 towards zero leprosy. As this study had some limitations regarding subject number and selection, a larger RCT study has been started to learn more about the breakthrough.

## Supporting information

S1 Data N/A: not available. Patient No. 4 did not show up for examination on 7 days and 3 months after injection.
(XLSX)

## References

[1] Vickers ER, Karsten E, Flood J, Lilischkis R. A preliminary report on stem cell therapy for neuropathic pain in humans. Journal of Pain Research 2014;7:255–63. doi: 10.2147/JPR.S63361 24855388 PMC4020887

[2] Granel B, Daumas A, Jouve E, Harlé JR, Nguyen PS, Chabannon C, et al. Safety, tolerability and potential efficacy of injection of autologous adipose-derived stromal vascular fraction in the fingers of patients with systemic sclerosis: an open-label phase I trial. Ann Rheum Dis 2015;74:2175–82. doi: 10.1136/annrheumdis-2014-205681 25114060 PMC4680117

[3] Bourin P, Bunnell BA, Casteilla L, Dominici M, Katz AJ, March KL, et al. Stromal cells from the adipose tissue-derived stromal vascular fraction and culture expanded adipose tissue-derived stromal. Cytotherapy 2013;15(6):641–8. 23570660 10.1016/j.jcyt.2013.02.006 PMC3979435

[4] Moon KC, Chung HY, Han SK, Jeong SH, Dhong ES. Possibility of Injecting Adipose-Derived Stromal Vascular Fraction Cells to Accelerate Microcirculation in Ischemic Diabetic Feet: A Pilot Study. International Journal of Stem Cells 2019;12(1):107–13. doi: 10.15283/ijsc18101 30836733 PMC6457712

[5] van Brakel WH, Sihombing B, Djarir H, Beise K, Kusumawardhani L, Yulihane R, dkk. Disability in people affected by leprosy: the role of impairment, activity, social participation, stigma and discrimination. Global Health Action. 2012; doi: 10.3402/gha.v5i0.18394 22826694 PMC3402069

[6] Truman RW, Andrews PK, Robbins NY, Adams LB, Krahenbuhl JL, Gillis TP. Enumeration of *Mycobacterium leprae* Using Real-Time PCR. PLoS Negl Trop Dis 2008; 2(11): e328. Retrieved from: doi: 10.1371/journal.pntd.0000328 18982056 PMC2570796

[7] van Brakel WH, Nicolls PG, Das L, Barkataki P, Suneetha SK, Jadhav R, dkk. The INFIR cohort study: investigating prediction, detection and pathogenesis of neuropathy and reactions in leprosy. Methods and baseline results of a cohort of multibacillary leprosy patients in North India. Lepr Rev. 2005;76:14–34. 15881033

[8] Schipper BM, Marra KG, Zhang W, Donnenberg AD, Rubin JP. Regional anatomic and age effects on cell function of human adipose-derived stem cells. Ann Plast Surg 2008;60(5):538–44. doi: 10.1097/SAP.0b013e3181723bbe 18434829 PMC4160894

[9] Vilaboa SDA, Navarro-Palou M, Llull R. Age influence on stromal vascular fraction cell yield obtained from human lipoaspirates. Cytotherapy 2014;0:1–6. 10.1016/j.jcyt.2014.02.007 24726656

[10] Strong AL, Burow ME, Gimble JM, Bunnell BA. The Effects of Obesity on Adipose-Derived Stromal Cells and Impact on Breast Cancer Tumorigenesis. J Cancer Biol Res 2014;2(1):1031.

[11] Silva KR, Côrtes I, Liechocki S, Carneiro JRI, Souza AAP, Borojevic R, et al. Characterization of stromal vascular fraction and adipose stem cells from subcutaneous, preperitoneal and visceral morbidly obese human adipose tissue depots. PLoS ONE 2017;12(3):e0174115. Retrieved from: doi: 10.1371/journal.pone.0174115 28323901 PMC5360317

[12] Baptista LS, Silva KR, Borojevic R. Obesity and weight loss could alter the properties of adipose stem cells? World J Stem Cells 2015;7(1):165–73. doi: 10.4252/wjsc.v7.i1.165 25621116 PMC4300927

[13] Bora P, Majumdar AS. Adipose tissue-derived stromal vascular fraction in regenerative medicine: a brief review on biology and translation. Stem Cell Research & Therapy 2017;8:145. doi: 10.1186/s13287-017-0598-y 28619097 PMC5472998

[14] Becton, Dickinson and Company BD Biosciences. Catalog No. 341071. CD45/CD34. Monoclonal Antibodies Detecting Human Antigens. Retrieved from: https://www.bd.com/resource.aspx?idx=1116(n.d.). Retrieved December 30, 2019, from https://www.bdbiosciences.com

[15] Ceceli E, Gumruk S, Okumus M, Kocaoglu S, Goksu H, Karagoz A. Comparison of 2 methods of neuropathic pain assessment in carpal tunnel syndrome and hand functions. Neurosciences (Riyadh). 2018;23(1):23–8. doi: 10.17712/nsj.2018.1.20170345 29455217 PMC6751908

[16] Margareta K. Validity and Reliability Test of Indonesian Version of PainDETECT Instrument to Identify Neuropathic Pain Components. [Thesis of Neurology Resident Education Program]. Jakarta: Medical Faculty of Universitas Indonesia; 2014.

[17] Miranville A, Heeschen C, Sengenès C, Curat CA, Busse R, Bouloumié A. Improvement of postnatal neovascularization by human adipose tissue-derived stem cells. Circulation. 2004 Jul 20;110(3):349–55. doi: 10.1161/01.CIR.0000135466.16823.D0 Epub 2004 Jul 6. . 15238461

[18] Karatan B, Akşam E, Erden E, Demirseren ME. Effects of adipose derived stromal vascular fraction on diabetic neuropathy: an experimental study. J Plast Surg Hand Surg. 2019 Dec;53(6):335–340. doi: 10.1080/2000656X.2019.1632205 Epub 2019 Jun 26. 31240978

[19] World Health Organization. Towards zero leprosy. Global leprosy (Hansen’s Disease) strategy 2021–2030. Geneva: World Health Organization; 2021.

